# Towards Tracking of Deep Brain Stimulation Electrodes Using an Integrated Magnetometer

**DOI:** 10.3390/s21082670

**Published:** 2021-04-10

**Authors:** Thomas Quirin, Corentin Féry, Dorian Vogel, Céline Vergne, Mathieu Sarracanie, Najat Salameh, Morgan Madec, Simone Hemm, Luc Hébrard, Joris Pascal

**Affiliations:** 1 Institute for Medical Engineering and Medical Informatics, School of Life Sciences, University of Applied Sciences and Arts Northwestern Switzerland (FHNW), 4132 Muttenz, Switzerland; corentin.fery@fhnw.ch (C.F.); dorian.vogel@fhnw.ch (D.V.); celine.vergne@fhnw.ch (C.V.); simone.hemm@fhnw.ch (S.H.); joris.pascal@fhnw.ch (J.P.); 2 Icube laboratory, UMR 7357 (University of Strasbourg/CNRS), 67412 Illkirch, France; morgan.madec@unistra.fr (M.M.); luc.hebrard@unistra.fr (L.H.); 3 Department of Biomedical Engineering, Linköping University, 581 83 Linköping, Sweden; 4 Center for Adaptable MRI Technology, Department of Biomedical Engineering, University of Basel, 4123 Allschwil, Switzerland; mathieu.sarracanie@unibas.ch (M.S.); najat.salameh@unibas.ch (N.S.)

**Keywords:** deep brain stimulation, magnetic field mapping, magnetic tracking system, three-dimensional magnetometer, image guided intervention

## Abstract

This paper presents a tracking system using magnetometers, possibly integrable in a deep brain stimulation (DBS) electrode. DBS is a treatment for movement disorders where the position of the implant is of prime importance. Positioning challenges during the surgery could be addressed thanks to a magnetic tracking. The system proposed in this paper, complementary to existing procedures, has been designed to bridge preoperative clinical imaging with DBS surgery, allowing the surgeon to increase his/her control on the implantation trajectory. Here the magnetic source required for tracking consists of three coils, and is experimentally mapped. This mapping has been performed with an in-house three-dimensional magnetic camera. The system demonstrates how magnetometers integrated directly at the tip of a DBS electrode, might improve treatment by monitoring the position during and after the surgery. The three-dimensional operation without line of sight has been demonstrated using a reference obtained with magnetic resonance imaging (MRI) of a simplified brain model. We observed experimentally a mean absolute error of 1.35 mm and an Euclidean error of 3.07 mm. Several areas of improvement to target errors below 1 mm are also discussed.

## 1. Introduction

Deep brain stimulation (DBS) is a neuro-surgical procedure used in routine treatment for several movement disorders like Parkinson’s disease and essential tremors [1]. The therapy consists in delivering electrical stimulation using multi-contact electrodes implanted in the structures of the brain responsible for the regulation of movement.

The anatomical structures targeted for electrical stimulation in the case of movement disorders have sizes in the range of millimeters, and minor errors in the targeting procedure can lead to massive differences in the symptomatic effect of stimulation [2]. One way to track the position of the electrode in relation to pre-operative imaging is using electrophysiology recordings to identify anatomy-specific patterns. This however requires the patient to be awake and comes with increased risks of complications, for instance higher hemorrhagic risks [3]. Another way would consist in using intraoperative imaging. The standard procedure relies on the use of X-ray imaging combined to stereotactic MRI. Recent development has tended to favor intraoperative MRI to achieve direct visualization [4] but this implies the availability of a specific operating theater. In addition, the electrode may move substantially during treatment, impacting significantly the patient outcome [5]. This is a consequence of the intentional flexibility of the electrode in order to avoid damaging the surrounding tissue would the brain be subject to motion. Considering the importance of electrode positioning in the brain, currently available solutions [6] are complex, costly and can be harmful for the patient. In this context, a magnetic tracking system, with sensors integrated directly in the electrode, allows tracking the position precisely during the surgery, as well as after the surgery.

In many biomedical applications optical tracking systems are used, for instance in computer-aided surgery [7]. However, optical tracking is intrinsically limited to applications where the line of sight is guaranteed. In DBS surgery, the electrode is occluded by the patient’s brain. In order to circumvent this problem, other approaches such as magnetic tracking could be used. Magnetic tracking systems are used in many medical applications [8], for instance in interventional MRI. In this case, the unique relationship between the magnetic field gradients and the position within an MRI bore enables the real-time localization of a magnetic sensor during an imaging sequence. A CMOS integrated three-dimensional Hall magnetometer, which has been integrated inside a surgical tool model for tracking purpose, has already been demonstrated [9]. Magnetic tracking is also used in electrophysiology procedures using commercially available solutions such as the Biosense Webster (Irvine, CA, USA) Carto3^®^ [10], or the Abbott (Chicago, IL, USA) EnSite Precision™ cardiac mapping systems [11]. These systems feature a magnetic field source, which generates magnetic fields varying in the range of 100 kHz. Miniaturized coils integrated within the catheter tip, pick up the signal necessary to determine the position of the catheter. Similarly, the NDI (Waterloo, ON, Canada) Aurora^®^ system is a customizable electromagnetic tracking system based on miniaturized coils for sub-millimeter applications [12].

A commercially available tracking system based on miniaturized coils has been recently investigated for DBS [13]. However, we believe that miniaturized coils are not the most promising technology for DBS. First, available micro-coils designed for magnetic tracking have sub-millimeter diameters but can reach up to one centimeter in length. These types of solenoid will then bend within the DBS electrodes which must be flexible. The bending affects their electromagnetic properties and thus reduces tracking precision. Second, micro-coils are sensitive to the rate of change of the magnetic flux (Φ), therefore to obtain a significant pick-up voltage at the output, the magnetic field used is typically varying around 100 kHz. This can be challenging in a DBS surgery environment since Eddy currents might occur in metallic parts with large surfaces located near the head of the patient, for instance in the stereotactic apparatus which guides the electrodes during implantation. The effect of eddy currents disturbs the magnetic field and consequently reduces accuracy. Moreover, the integration of multiple micro-coils along a DBS electrode, which would allow the surgeon to monitor the DBS electrode orientation and bending, requires two wires per coil which results in a high microsystem engineering complexity. In contrast, integrated three-dimensional magnetometers have been recently introduced on the market in thin sub-millimeter quadratic packages, such as wafer-level chip scale packages (WLCSPs) [14]. Besides, integrated magnetometers using three-dimensional Hall effect sensors are immune to mechanical stress thanks to the spinning current method [15]. This technique implemented with on-chip circuitry cancels the sensor offset due to mechanical stress applied on the silicon die. It also reduces the sensor noise, increasing its resolution [16].

In addition to their small size and their immunity to mechanical stress, it is important to note that integrated magnetometers usually feature a digital output with a serial communication bus such as the two wires interface (TWI), or the serial protocol interface (SPI). This makes it possible to connect a daisy chain of sensors along the same communication bus, which reduces the number of wires. Using a single TWI bus, it is possible to connect up to 128 sensors in series using only four connections in total: two wires for the communication and two wires for the power supply. This facilitates the integration of integrated magnetometers within a DBS electrode. Finally, magnetic tracking is a non-ionizing method, which contrasts with localization based on X-ray scan. Therefore, magnetic tracking using integrated magnetometers is to our knowledge the most promising solution for DBS.

The level of miniaturization as well as the sensitivity performances of monolithic integrated magnetometers reached by the semiconductor industry during the last decade make it possible to design various surgical tools instrumented with magnetometers [17,18]. These magnetometers are based on several physical principles, mainly the Hall effect, or the magnetoresistance, and address among others the automotive and consumer electronics markets. The typical applications are position sensing in automotive and electronic compass in smartphones. However, after calibration, it has been demonstrated that these sensors are capable to reach the accuracy level required in various medical applications [19,20].

Most of the magnetic tracking systems are composed of a magnetic field source generating magnetic field lines of known intensity and geometry, associated with one or several magnetometers located within the tracked object. Alternatively, a miniaturized magnetic source, typically a small permanent magnet, can be integrated within the tracked object whereas an arrangement of magnetometers measures externally the generated field to calculate the position of the object [21,22]. In deep brain stimulation surgery, the sub-millimeter inner diameter of the electrodes allows one to integrate only a tiny magnet which would generate field intensities below 10 nT outside the head of the patient. This makes it challenging to measure with a satisfying signal to noise ratio when using standard miniaturized sensors such as magneto-resistive and Hall effect sensors. Besides, the more sensitive fluxgate sensors which offer around 1 nT resolution [23] are not good candidates for the DBS application due to their bulky dimensions and high cost.

In this paper, we developed a model of DBS electrode which core technology relies on a three-dimensional Hall sensor and which performances have been evaluated for tracking purpose. Section 2 describes the magnetic source, the properties of the magnetometer integrated within the DBS electrode model as well as the localization algorithm. Section 3 explains the experimental results obtained by comparing the system with both a visual tracking and an MR-imaging. A discussion and perspectives are proposed in Section 4, followed by conclusions in Section 5.

## 2. System and Method

**Figure 1:**
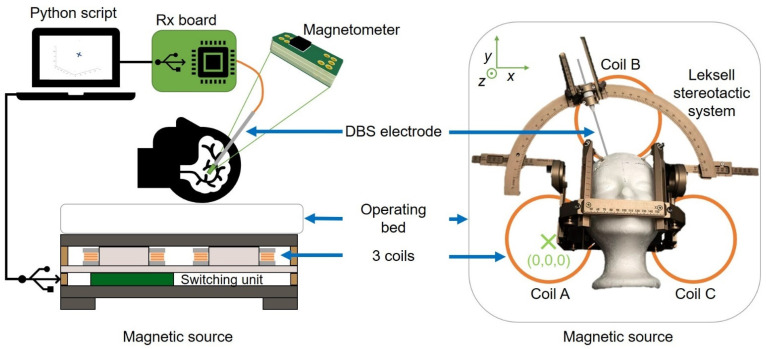
Scheme of the magnetic tracking system in the DBS context. Patient lying on the operating bed with the magnetic source located underneath the head (**left**). Top view illustrating the position of the Leksell’s stereotactic guiding apparatus (**right**).

The appropriate setup for the implementation of a magnetic tracking system for DBS electrodes consists of at least one sub-millimeter magnetometer chip fitted inside the electrode and associated to a magnetic field generator located for instance underneath the head of the patient. Figure 1 gives an overview of the magnetic tracking system for DBS described in this paper. It depicts the magnetic source in cross-section and in top view with the head of the patient located above the source. Figure 1 also shows the stereotactic guiding apparatus from Leksell (Elekta, Sweden), which is used to guide the DBS electrodes. This apparatus contains ferromagnetic materials which disturbs locally the magnetic field. However, the system described in this paper compensates for the field distortions as explained in Section 2.2. The sensing and control electronics and software is also illustrated in Figure 1.

### 2.1. The Magnetic Source

**Figure 2:**
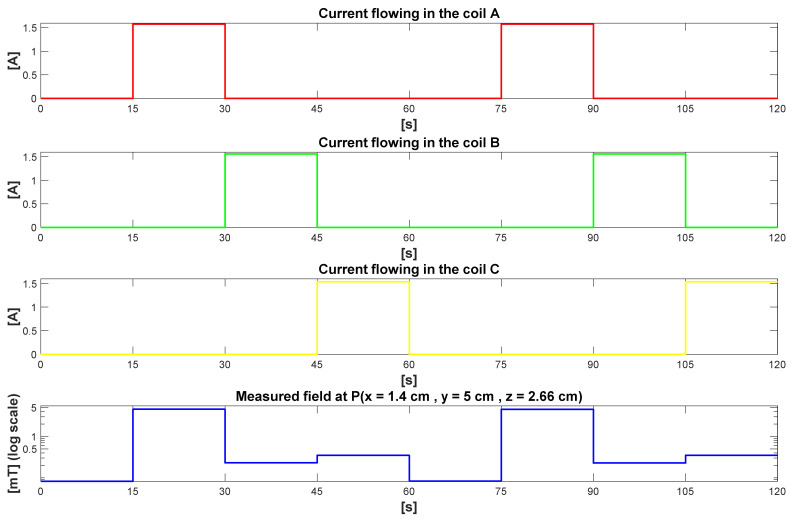
Sequences of the current flowing through the coils A, B and C, and the corresponding measured magnetic field intensity at a location point P (x = 1.4 cm, y = 5 cm, z = 2.66 cm).

Three coils arranged in a triangle (Figure 1, right) generate sequentially three different magnetic fields and allow for localization using a trilateration algorithm [24]. To control sequentially these three coils, a switching unit (Figure 1, left), featuring a microcontroller unit, MCU and MOSFET transistors was developed. This unit controls dynamically the current flow through the coils, and its synchronization with the magnetometer acquisitions is ensured by the control software running on a standard PC (Figure 1, left). Three phases of 15 s followed by a fourth phase of 15 s without any current flowing through any of the coils form the sequence operated continuously by the source. During the fourth phase the algorithm can update the offset values of the sensors as well as any perturbing fields such as the Earth magnetic field as described later in Section 2.4. Figure 2 shows the sequence of the current flowing through the coils and the corresponding magnetic field intensity at a pre-defined location point P (x = 1.4 cm, y = 5 cm, z = 2.66 cm).

The magnetic field source requires two cables for respectively connecting to a power supply and to a PC USB port. The three coils and the switching unit forming the magnetic field source are packaged in a 50 cm × 50 cm × 13.5 cm housing. The overall setup makes the installation and removal of the source underneath the head of the patient rather simple, prior and after surgery. The system is therefore adaptable to different operating theater configurations, making it possible to adapt to almost every hospital.

### 2.2. The Magnetic Field Map

Conventional tracking systems usually implement a theoretical map of the magnetic field generated by the source. Developing a computational model of the magnetic field lines [25], derived from the Biot-Savart law [26], is required for such an approach. The accuracy of the system depends in this case on the accuracy of the model of the magnetic field. This method becomes increasingly complex and less accurate when the source exhibits high manufacturing tolerances, or when a distortion of the magnetic field lines is induced by perturbing ferromagnetic objects located near the object to be tracked. In this case a satisfying computational model is challenging to obtain and, in any case, specific to a given source setup and source environment.

**Figure 3:**
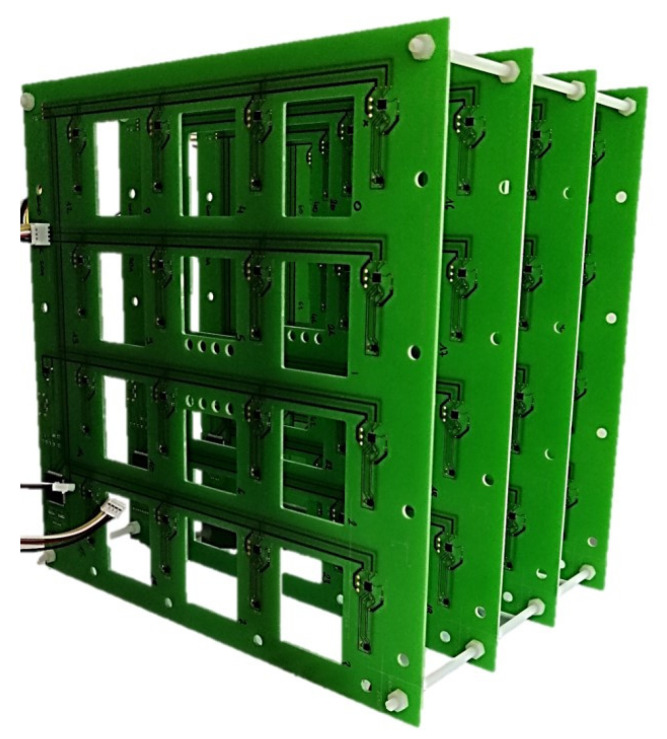
Magnetic field camera developed to experimentally map the magnetic field generated by the source. An arrangement of 4 × 4 × 4 = 64 magnetometer chips, separated by 48 mm in the X, Y and Z directions, provides the 64 magnetic field intensity values which are then interpolated to obtain a fine map of the field.

To address this issue, we propose to use an experimental map of the magnetic field. An in-house magnetic camera like the one presented in [27] has been developed. This device measures the full field vector information synchronously at 64 locations in the volume of interest. Figure 3 shows the camera which is a cubic arrangement of 4 × 4 × 4 = 64 magnetometers mounted on four printed circuit boards (PCBs).

The PCB manufacturing process allows one to obtain a position of the magnetometers with a spatial resolution of less than 100 µm, which is significantly below the targeted accuracy of one millimeter for the localization of DBS electrodes. The 64 magnetometers measurements are interpolated with a tri-cubic interpolation to finely model the magnetic field. Using this approach, the accuracy of the map depends on the number of sensors in the camera, on the accuracy of these sensors and finally on the capability of the interpolation to model magnetic fields lines properly. In the future a finite element method (FEM) based on a physical model of the magnetic field [28] or a 3D cellular nonlinear network (CNN) [29] will replace the mathematical interpolation in order to improve the accuracy of the map.

The map can be performed in the presence of perturbing ferromagnetic objects such as the stereotactic guiding apparatus of the DBS electrodes which can contain ferromagnetic parts. In this case, the magnetic field camera is placed where the patient’s head should be located, and in this manner, the distortion of the magnetic field lines geometry is included in the map. It is important to note that a distortion of the magnetic field lines is an issue for a tracking system using a theoretical map. In contrast, using an experimental map, the accuracy of the tracking will not be affected since the distortion is included in the model. The unique relationship between the position and the magnetic field is still valid in the case of a geometrically distorted magnetic field. The electrode itself is not ferromagnetic. Therefore, an accurate tracking system does not require an ideal, non-distorted field map. A dynamic compensation of magnetic field distortions [30,31] is neither required for DBS since the operating theater can be protected from magnetic field perturbations. Such a protection can be implemented by an arrangement of magnetometers placed around the head of the patient which checks the integrity of the magnetic field continuously during surgery. The presented system requires an accurate characterization of the field map which is performed one single time with the magnetic camera prior to surgery. Four maps of the magnetic fields are acquired which correspond to the four magnetic fields generated by the source during each of the four phases illustrated in Figure 2. The four maps are interpolated and stored on the PC for their use in the localization algorithm described in Section 2.4.

### 2.3. Magnetometer Selection and Design of the Sensor Electronics for DBS Electrodes

Most of the tracking systems available on the market use micro coils as sensing elements. However, such systems can also be realized with integrated three-dimensional magnetometers, for instance with Hall effect sensors or magnetoresistive sensors. These types of magnetometers, also called integrated compass, allow measuring maximal magnetic field strength up to several mT like the field generated by the source presented in Section 2.1.

We have selected a Hall effect sensor for the integration within a model of DBS electrode. Best in class commercially available Hall sensors exhibit high resolutions, typically 1 µT per least significant bit (LSB), and moderate noise level, typically less than 10 µT rms. Moreover, they often cost only around one USD per sample. Besides, they are miniaturized while integrated in sub-millimeter packages. The measuring range of most of the commercially available integrated Hall magnetometers featuring both the Hall sensing element and its conditioning electronics reaches few tens of mT. This moderate range is not resulting from the Hall effect itself, which induces no saturation effect and shows a good linearity up to approximately 3 T [32]. This is a consequence of the integrated amplifying chain which is usually designed to amplify the Hall voltage induced by the exposure to small permanent magnets. Therefore, most of the integrated Hall magnetometers will saturate when they are exposed to strong fields such as within an MRI scanner, typically operating at 3 T. However, even if no measurements can be performed in strong fields using most of the Hall magnetometers, they will not be destructed by the exposure to strong fields and can operate again when they are removed and placed back in a low field environment remaining below their measuring range. For this reason, the integrated magnetometers based on Hall effect are resilient to MRI environment. This is not the case of integrated magnetometers based on magnetoresistances which would be permanently destroyed by an exposure to a 3 T magnetic field. This is due to the presence of a soft magnetic layer in the stack of a magnetoresistance structure which is used to sense the magnetic field [17]. Under an exposure to a strong magnetic field the magnetization of this layer would be permanently pinned, which destructs the sensing capability of the magnetoresistance. The use of a Hall magnetometer allows us to validate the capability of the system to operate in three-dimensions and without line of sight by comparing the calculated chip location with its position provided by an MRI image as described in Section 3.4. For possible future clinical use, the choice of a Hall effect magnetometer will not prevent the patients, instrumented with the proposed smart DBS electrodes, to undergo an MRI.

We have selected a magnetometer from Asahi Kasei Microdevices (Tokyo, Japan), the AK9970 [33]. It can measure a range of ±36 mT and exhibits a resolution of 1.1 µT/LSB, as well as a noise level of 5 µT rms. It features an integrated 16 bits analog to digital converter (ADC). The maximal output data rate reaches 100 Hz, enabling averaging up to one hundred averaging per operating phase of the magnetic source presented in Section 2.1. The chip is encapsulated in a wafer-level chip-scale, WLCSP package of 1.35 mm × 1.35 mm × 0.57 mm. This is slightly too large for an integration into DBS electrodes which have an outer diameter ranging from 1.27 to 1.41 mm [34]. However, this size is satisfying for this feasibility study where we use the model of a DBS electrode of larger dimensions than clinical electrodes. An integration of a similar sensor in a sub-millimeter package of typically 750 µm × 750 µm × 500 µm is a standard task for the microelectronics industry [14]. Such a smaller package shall be used for possible future clinical smart DBS electrodes development.

A single AK09970D chip has been assembled on a PCB (Figure 1). The number of wires connecting to this PCB is minimal and corresponds to the two wires required for the serial communication bus, a general-purpose input output (GPIO) wire, as well as two wires for the power supply. To keep the prototyping easy, the PCB dimensions of the first demonstrator presented in this paper are still relatively large: 9 mm × 2.3 mm × 1 mm. Nevertheless, it allows us to integrate the PCB in a model of DBS electrode presented later in Section 3.2.

### 2.4. The Localization Algorithm

To locate the magnetometer a trilateration algorithm [24] is implemented in the software. Unlike triangulation, which uses distances and angles between the magnetometer and the triangular source, the trilateration algorithm uses only the distances. After loading the three magnetic fields maps, the software sequentially activates the three coils to get three values of magnetic field strength measured by the magnetometer during each phase of the source activation sequence. The fourth phase is used to subtract the offset of the magnetometer, the earth magnetic field, and any other magnetic field perturbation such as the presence of small magnets near the magnetometer. To estimate the location of the magnetometer, the norm of the relative difference between the magnetic field strength obtained with the magnetometer’s values and the field map is minimized. It is necessary to work with relative differences because the magnetic field strength measured at different locations within the volume of interest can change with several orders of magnitude. Finding the minimum of the following function *f* allows one to find the position *X* of the magnetometer:

(1) $$f\left(X^{\prime }\right)=\;{\left\|\begin{array}{c} \frac{B_{A}\left(X\right)-\;B_{A}^{r}\left(X^{\prime }\right)}{B_{A}\left(X\right)}\\ \frac{B_{B}\left(X\right)-\;B_{B}^{r}\left(X^{\prime }\right)}{B_{B}\left(X\right)}\\ \frac{B_{C}\left(X\right)-\;B_{C}^{r}\left(X^{\prime }\right)}{B_{C}\left(X\right)} \end{array}\right\|}_{2}$$

where $X^{\prime }={\left(\begin{array}{ccc} x^{\prime } & y^{\prime } & z^{\prime } \end{array}\right)}^{T}$ is the position variable of the function, $X=\;{\left(\begin{array}{ccc} x & y & z \end{array}\right)}^{T}\;$ is the real and unknown position of the magnetometer, $B_{i}$ is the value of the measured magnetic field strength of coil *_i_*, and $B_{i}^{r}$ is the value of the mapped magnetic field strength of coil *_i_*.

To minimize this function *f*, a quasi-Newton method is used: the extended version of the Broyden–Fletcher–Goldfarb–Shanno algorithm, the L-BFGS-B algorithm.

The software synchronizes the acquisition of the magnetic field values performed by the magnetometer with the magnetic field generation performed by the source. Several acquisitions are performed during each phase of the source sequence and averaged. This increases the accuracy, and a trade-off between refresh rate of the calculated positions and accuracy has to be done. In the DBS application a high-speed tracking is not required, and a refresh rate of 60 s is satisfying. In this paper results are presented with an averaging of hundred magnetometer data obtained during each of the four phases. Since each of the four phases lasts 15 s, we obtain a refresh rate of approximately 61 s. This corresponds to the four phases and an additional one second necessary for the computation.

### 2.5. Patient Exposure to Magnetic Field

The International Commission on Non-Ionizing Radiation Protection (ICNIRP) provides recommendations to estimate and limit the patient exposure to magnetic fields [35]. Working at low magnetic field and low frequency reduces the patient exposure. The magnetic source used in this work consists of three coils through which a continuous current is injected sequentially every 15 s. A fourth phase where no current is flowing is also included in the sequence for offset compensation purpose. This results in low frequency magnetic field pulses settling in the volume of interest as illustrated in Figure 2.

In the presented configuration the magnetic source generates a maximum field intensity of 5.2 mT in the volume of interest where the head of the patient shall be located. The patient exposure to magnetic fields has been calculated for the position where the magnetic field has the shape illustrated in Figure 2. The ICNIRP guideline recommends the calculation of an exposure coefficient, which is based on a spectral decomposition of the field. This coefficient corresponds to the sum of the ratios between the magnetic field value at each harmonic and a reference level calculated for the same frequency.

According to the guideline only harmonics above 1 Hz shall be considered. With the maximal field strength generated for this work (see Figure 2); the calculated exposure coefficient is 0.50. This is less than 1, the maximum recommended value. To calculate this value, we have considered the first 250 harmonics of the signal above 1 Hz. This value is very conservative since it is easy to suppress the high harmonics of the rectangular magnetic field by applying a simple low pass filter to the coils’ activation currents.

Towards future applications of the presented tracking system where the refresh rate shall be for instance 2 Hz, we still have calculated an exposure coefficient below 1 (0.73). The first ten harmonics of a 2 Hz signal with the same shape as the one depicted in Figure 2 have been considered. This corresponds to a configuration where the rectangular waveform of the magnetic field is strongly filtered through a low pass filtering of the coils’ activation currents.

### 2.6. System Validation Experiments

**Figure 4:**
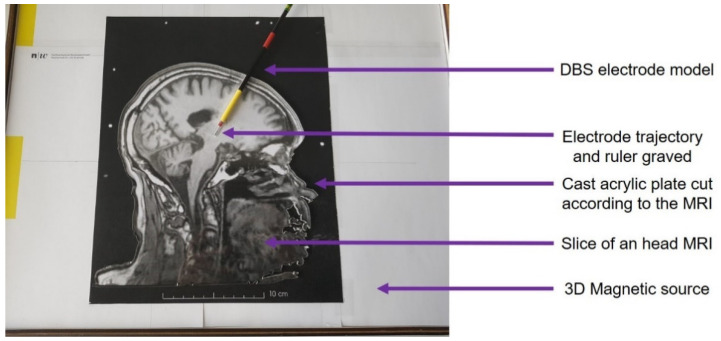
Experimental setup: the DBS electrode model is inserted along an engraved trajectory. A visual assessment of the position is then performed at each tested location.

To validate our system, an estimation of the accuracy and a functional validation while tracking without line of sight are required. In a first experiment, a visual method was selected as reference measurement for the estimation of the accuracy. Starting from an MR image of a patient with the DBS electrode implanted, a cast acrylic plate was cut with a laser cutter (Trotec, Marchtrenk, Austria) according to the outline of the head and the DBS electrode trajectory (see Figure 4). A millimeter precision ruler has been engraved, also by laser, along the implantation trajectory for an easy control of the real position of the electrode model (Figure 4). In this manner a visual reading of the real position of the electrode provides a reference with an accuracy below one millimeter.

The two-dimensional brain model consists in the acrylic plate aligned on a printout of the MR image at the 1:1 scale. This model was then placed over the three-dimensional magnetic source (Figure 4). The DBS electrode model was inserted in the engraved electrode trajectory.

In a second experiment, an assessment of the magnetic tracking system in three dimensions and without line of sight, was performed in a low field MRI (100 mT) on a simplified brain model consisting in a watermelon, which dimensions and shape are close to those of a human head. The DBS electrode model was inserted in the watermelon and its trajectory was extracted by using its voxel positions, easily identifiable from hypointense regions.

**Figure 5:**
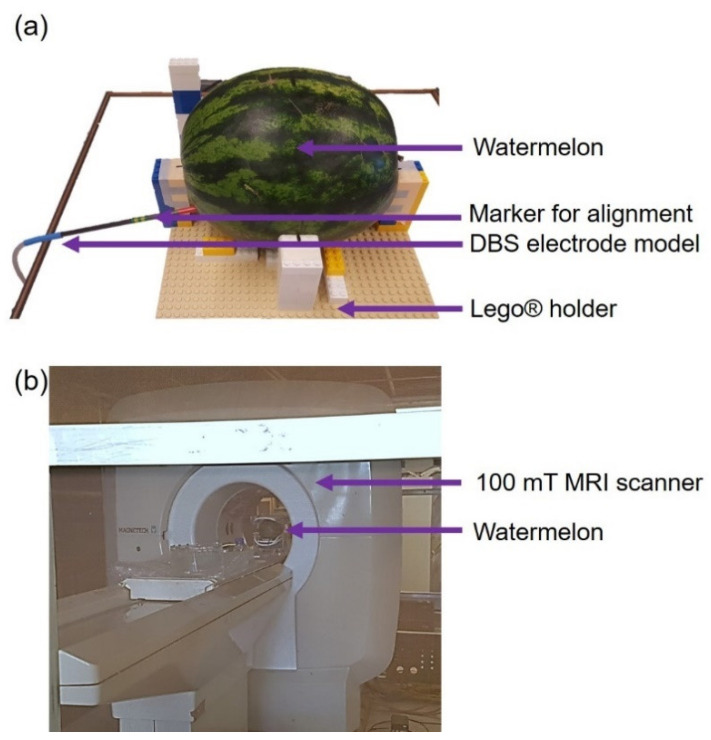
(**a**) Experimental setup: the DBS electrode model is inserted in a watermelon. The watermelon is then placed on a Lego^®^ holder on top of the tracking magnetic source. (**b**) 100 mT MRI scanner used to image the watermelon with the inserted electrode.

Figure 5 shows the experimental setup and the 100 mT MRI scanner used for imaging (MAG-1000, Magnetech, Vandoeuvre-lès-Nancy, France). The setup includes the watermelon in which the DBS electrode model was inserted at different depths. The latter were measured each time using the tracking system outside the MRI bore, and images were sequentially acquired with the model placed within the MRI scanner. A constant step of 5 cm was chosen between the different positions. A set of Lego^®^ bricks is used to easily reposition, after each MRI, the watermelon exactly at the same place and orientation with respect to the magnetic tracking source. Markers drawn at four locations both on the watermelon and on the Lego^®^ bricks ensured the alignment during repositioning with an estimated spatial resolution below one millimeter.

A head antenna together with a three-dimensional gradient echo sequence were used with 60 signal averages and an acquisition matrix of 96 × 96 × 29, resulting in a voxel size of 2.4 mm × 1.9 mm × 5.5 mm. Four positions were measured for the following insertion depths: 0 cm, 5 cm, 10 cm, and 15 cm. The trajectory can be considered linear thanks to the high rigidity of the carbon tube used for the DBS electrode model.

## 3. Results

### 3.1. Magnetic Source Characterization

A characterization of the magnetic source was done with the magnetic field camera presented in Section 2.2. At each location of the (16.8 cm × 11.2 cm × 12.2 cm)-volume of interest, three different magnetic field values were sequentially acquired with different intensities according to the relative distance to the coils. This map was performed without the stereotactic guiding apparatus. This volume could easily be increased by performing a larger map for instance to fully cover the patient head.

**Figure 6:**
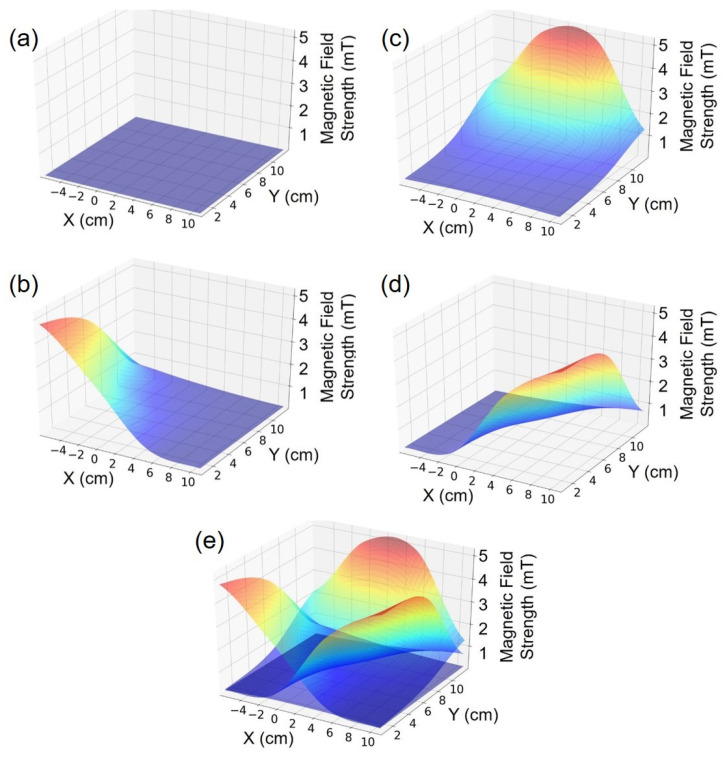
(**a**) Magnetic field intensity map obtained by the tri-cubic interpolation of experimental mapping data when all coils are turned off. (**b**–**d**) Magnetic field intensity maps obtained by the tri-cubic interpolation of mapping experimental data when the coils A, B and C are respectively turned on. (**e**) Superimposition of (**b**–**d**) maps to illustrate the trilateration principle.

Figure 6 shows the four maps measured by the magnetic field camera after interpolation and for each of the three coils (b) (c) (d) as well as the offset map (a). The maps are displayed in the X-Y plane for an arbitrary Z-coordinate fixed at Z = 2.16 cm. A superimposition of the three maps (e) illustrates the trilateration principle, where each point within the volume of interest corresponds to a unique combination of three field strength values.

### 3.2. Design and Fabrication of a DBS Electrode Model Integrating a Three-Dimensional Magnetometer

**Figure 7:**
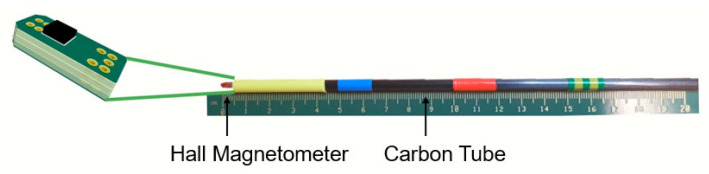
DBS electrode model with the integrated three-dimensional Hall magnetometer. The carbon tube has a diameter of 5 mm. This version has been used for the experimental accuracy estimation of the tracking system.

**Figure 8:**
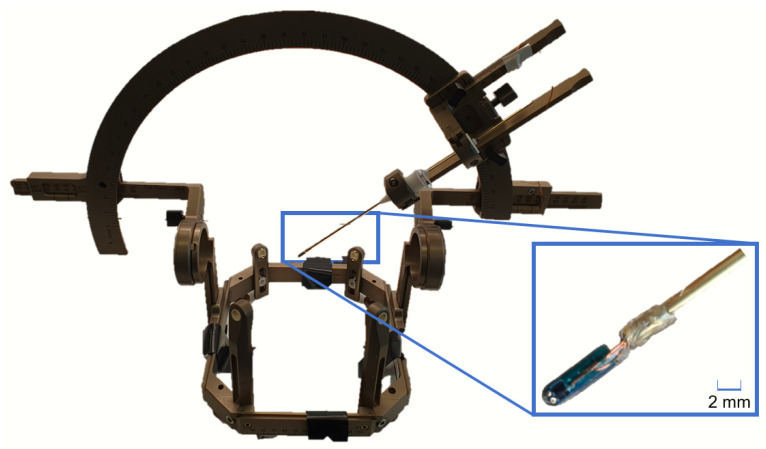
Miniaturized version of the DBS electrode model with the integrated three-dimensional Hall magnetometer, mounted on a Leskell system. The tip of the electrode has a diameter of 2 mm.

For the sake of simplicity, an inexpensive model of DBS electrode which is larger than a real electrode has been designed and fabricated (Figure 7). This model of DBS electrode integrates a three-dimensional magnetometer to evaluate the feasibility of magnetic tracking for DBS. The magnetometer electronics has been integrated at the tip of a rigid carbon tube of 5 mm diameter. A second functional electrode model with only 2 mm diameter is depicted in Figure 8.

This miniaturized version features the same magnetometer chip. However, due to the higher rigidity of the 5 mm diameter version, we used the 5 mm version for the accuracy measurements where the electrode model needs to be inserted through a watermelon. For both versions the tube and electronics are non-ferromagnetic and therefore do not disturb the magnetic field applied for tracking. It is also MRI-safe. This will allow us to evaluate the system performances compared to a tracking of the electrode based on MR images. The probe electronics consists of a PCB on which the three-dimensional magnetometer (AKM 09970D) is mounted. This PCB is connected to a receiving board Rx through five connection cables of 150 µm diameter each. The Rx board is based on a microcontroller that reads out the magnetometer data. The Python software synchronizes the data acquisition with the activation of the magnetic field source through a pair of USB connections (Figure 1).

### 3.3. Accuracy Evaluation with Line of Sight

**Figure 9:**
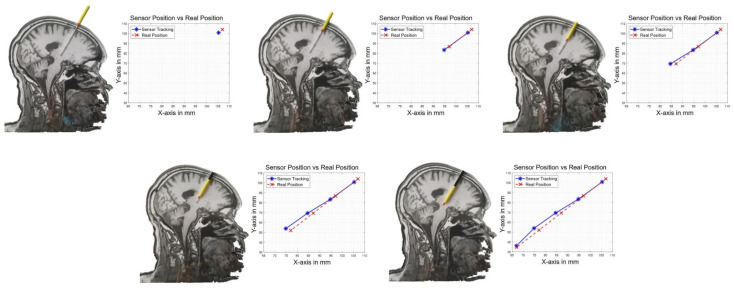
Experimental setup: the DBS electrode model is inserted along the engraved trajectory. The plots represent the reference linear trajectory in red vs. the trajectory estimated by the magnetic tracking system in blue for a Z-level of 2.66 cm.

According to the first experiment described in Section 2.6, we present in this section a set of measurements corresponding to five different positions acquired three times each along the linear trajectory of the electrode. The five positions have been defined at 0, 2, 4, 6 and 8 cm from the entry point to the final position of the tip of the electrode. Measured positions obtained with the magnetic tracking system are compared relatively to these five positions along the trajectory. The results are given in the X-Y plane for a constant Z level of 2.66 mm, meaning that the experiment has been performed with the two-dimensional brain model oriented parallel to the plane of the magnetic source. More experiments have been performed with various orientations of the brain model with respect to the source and gave similar results. Figure 9 gives an overview of the setup and the evaluation of the tracking accuracy, and Table 1 the corresponding values.

The magnetic tracking exhibits a mean absolute error of 1.76 mm along the X-axis, 2.00 mm along the Y-axis and 0.30 mm along the Z-axis (Table 1). The maximal absolute error distance is 2.47 mm along the X-axis, 3.28 mm along the Y-axis and 1.30 mm along the Z-axis for an insertion along the trajectory presented in Figure 9 (Table 1). Based on this experiment, the resulting mean absolute error (MAE) on the tracking coordinates in this measurement series is 1.35 mm which is close to the diameter of a real DBS electrode (~1.40 mm). The mean Euclidean error for the positions measured in this experiment is 3.07 mm (3.07 ± 0.905 mm with a confidence of 1 σ). The measurement uncertainty of the reference in this experiment is mainly introduced by the visual reading of the position. We can estimate that the reading introduces an uncertainty of about half a graduation that is ±0.5 mm.

### 3.4. Functional Assessment in Three Dimensions and without Line of Sight with MR Imaging of a Simple Brain Model

**Figure 10:**
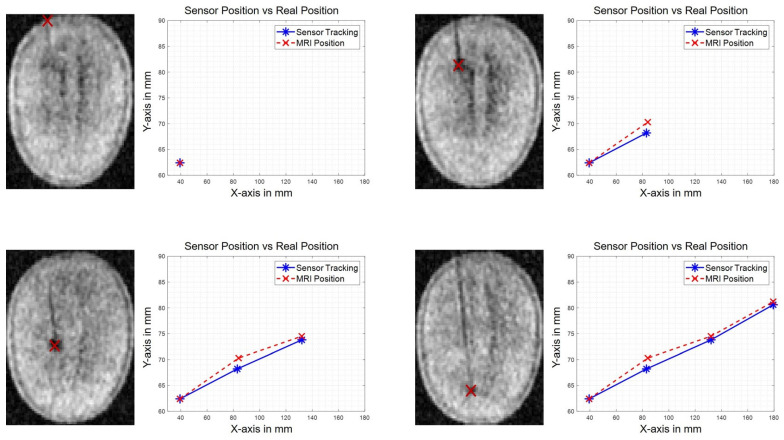
MR images of the watermelon with the DBS electrode model inserted. Position of the tip of the electrode measured on MR images (in red) vs. measured position obtained with the proposed tracking system (in blue).

Figure 10 shows the measured positions obtained with both the tracking system and MR images, as explained in Section 2.6 for the second experiment we performed. The results given in Figure 10 are in the X-Y plane for different MRI slices (Z-level) corresponding to four different positions of the electrode.

The data show a discrepancy between the positions obtained by magnetic tracking and those obtained on the MR images (Table 2). The MR image has a large voxel size of 5.24 mm along the Z-axis and can therefore not be considered as a reference for the estimation of the position error. This experiment solely demonstrates the capability of the tracking system to navigate in 3D. Therefore, we do not calculate here any error but simply the distance between magnetic tracking and MR images. The mean absolute distance is 0.52 mm along the X-axis, 1.48 mm along the Y-axis and 5.10 mm along the Z-axis. The data show a maximal absolute distance of 0.9 mm along the X-axis, 2.06 mm along the Y-axis and 8.94 mm along the Z-axis (Figure 10). The mean Euclidean distance measured in this experiment is 5.78 mm (5.78 ± 3.27 mm with a confidence of 1 σ).

The measurement uncertainty in this experiment is mainly introduced by the repositioning of the watermelon at each image. The repositioning is performed with a visual alignment on a marker and the uncertainty is therefore estimated to be ±0.5 mm.

## 4. Discussion

The feasibility study described in this paper opens the way to the development of instrumented DBS electrodes integrating miniaturized magnetometers for tracking purpose. Further miniaturization of the present electronics is needed to fit within clinical DBS electrodes. This can be done using available magnetometers packaged in sub-millimeter wafer level chip scale packages, which only increases manufacturing cost compared to the electrode models presented in this paper.

The magnetic source of the present tracking system is activating sequentially three coils with a dc current. This induces the occurrence of three successive continuous magnetic fields during each of the three phases. The tracking system uses an experimental mapping of the magnetic field intensity. The mapping is obtained with an in-house dedicated magnetic field camera. In this way the manufacturing tolerances of the magnetic source has no impact on the accuracy of the tracking. The introduction of a fourth phase in the sequential activation of the three coils, where no current is injected in any of the coils, allows the algorithm to compensate for the offset of the magnetometers, both during the mapping of the source and during the tracking. During this fourth phase the algorithm also compensates for possible external perturbations such as externally applied magnetic fields or the earth magnetic field. The system also compensates for the presence of ferromagnetic parts in the volume of interest if the mapping is performed in a configuration where these ferromagnetic parts, for instance the DBS electrodes navigation apparatus, are present. This is one of the crucial advantages of the presented tracking method for DBS surgery. The magnetic camera is therefore a key element directly influencing the tracking accuracy.

In order to improve the system accuracy, a calibration of each magnetometer of the magnetic camera within a large three-dimensional Helmholtz coil should be valuable, and shall be performed for further developments. Similarly, the magnetometer integrated at the tip of the electrode shall also be calibrated to improve the accuracy performances. Calibration means here the correction of the magnetometer gain expressed in least significant bits per Tesla, LSB/T. The offsets of the magnetometers do not need to be extracted during their calibration since their compensation is continuously ensured during the zero-current phase of the source sequence i.e., during its fourth phase. Increasing the number of magnetometers in the magnetic field camera, their resolution, and the accuracy of their position on the PCB shall also be investigated. Finally, using alternating currents to bias the coils would significantly reduce the power dissipation and allow one to achieve the same field intensity with a sinusoidal shape instead of a continuous one. The processing of the magnetometer data can be done in this case by the computation of a discrete Fourier transformation, DFT instead of using the averaging method presently used for continuous fields, and should also help in improving the magnetometer resolution since the measurement is determined over a very narrow bandwidth, i.e., selecting the fundamental spectral line. From the hardware point of view, alternating currents can easily be injected through a coil by tuning its impedance with the adequate capacitor to obtain a resonant LC-circuit. The implementation of all these system improvements should lead to a residual Euclidean error of less than 1 mm on the estimated electrode position.

The present mapping of the magnetic field corresponds to a volume of 16.8 × 11.2 × 12.2 cm^3^. The trajectory of the electrode demonstrated in Section 3 fits within this volume. However, a larger volume can easily be achieved using the same magnetic field camera. For instance, to fully cover the head of the patient plus some margin around, a mapped volume of 30 cm^3^ is recommended.

The refresh rate of the electrode location is around 61 s. The focus of this study was given on the estimation of the localization error. For this purpose, the sensor output data rate was set to a low value of 6.25 Hz to minimize the noise level. However, subsequent testing showed that the sensor exhibits the same rms noise value of 20 µT at 6.25 Hz and 100 Hz output data rate. This indicates that our system could also operate with 100 Hz sensor data rate. With this configuration the refresh rate of the localization would be below one second and would allow a dynamic localization of the DBS electrode. In this case, the amount of data provided by the magnetometers will be the same during each sequence of the source and therefore the signal-to-noise ratio will remain unchanged.

Accuracy has been characterized visually with a simple graduation engraved along the trajectory of the electrode, which is enough for an accuracy around one millimeter. The system exhibits a maximal absolute error of 3.28 mm, a mean absolute error of 1.35 mm, and a mean Euclidean error of 3.07 mm. This is promising for the application of tracking DBS electrodes. For further developments where the accuracy shall be improved to less than one millimeter, another reference tracking method is required. For instance, an infrared tracking system, such as the Optotrak Certus^®^ (NDI) [36] with a resolution of 10 µm can be used.

The validation of the tracking system without line of sight and in three dimensions has been successfully performed by comparing the measured location with the images obtained in a low field 100 mT MRI scanner. The voxel positions are known with certainty. The mean differences between the voxel position and the measured position in the X-Y plane were at most 1 mm (on the order of the tracking system error) but exceeded 5 mm in the Z direction where the voxel size is larger than 5 mm. This difference on Z-axis could be due to partial volume effects coming from the MR image resolution in the Z dimension. Therefore, despite relatively large voxel sizes it is useful to compare the positions obtained with the magnetic tracking and the one obtained by MR images. As the errors are smaller than the voxel size on each axis, it demonstrates the capability of the proposed tracking system to target the right voxel and thus to operate without line of sight and in three-dimensions.

The presented model of DBS electrode uses a Hall magnetometer that can be operated in a large range of magnetic field strengths. Despite being MR-safe, we chose to perform our measurements in a low-field 100 mT MRI scanner for two reasons. First, this allows repeating the validation procedure with possible future demonstrators which core technology is made of magnetoresistive magnetometers instead of Hall magnetometers. Even though magnetoresistive sensors outperform Hall sensors in terms of noise level, they are suffering permanent damage when placed in intense magnetic fields like found in conventional clinical scanners e.g., 3 T. Second, low field MRI is less sensitive to magnetic susceptibility changes, making the probe localization artifact-free, hence more accurate. Future development for better performance comparisons would include the use of more advanced imaging sequence in order to improve the spatial resolution.

The most significant improvement of the presented system would be the capability of measuring the orientation of the tip of the electrode in addition to its position. Indeed, the rotation of a DBS electrode around its axis modifies the distribution of the voltage applied to the brain tissues due to the geometry of the electrode’s metallization. That is the reason why there is a strong clinical interest to also track the orientation of the electrode [37]. Tracking the orientation of the electrode requires either to use a three-dimensional magnetic source or to integrate at least three magnetometers along the electrode. The second solution also offers the possibility to measure the bending of the electrode, that is another relevant parameter during and after implantation surgery.

Finally, a further outlook of the proposed tracking system is to monitor at regular intervals the position, the orientation, and the bending of the DBS electrodes after surgery and during daily life of the patient. After placing the magnetic source underneath the head of the patient, at the same location as during the surgery, the tracking procedure can be quickly performed by a physician without discomfort for the patient.

## 5. Conclusions

The magnetic tracking principle is already used in several surgical procedures but has not yet been implemented for DBS surgery even though the location of the electrodes is of prior importance for the treatment efficacy. This paper opens the way to the integration of magnetometers at the tip of DBS electrodes for tracking purpose. The presented tracking system can operate in combination with the DBS stereotactic guiding apparatus and the preoperative clinical imaging. The guiding apparatus helps to precisely position the entry point of the electrode, whereas preoperative imaging is used to determine the optimal electrode location to maximize the treatment effect. The magnetic tracking system has been developed to make the link between these two elements of the surgical procedure by allowing the surgeon to have the full control of the implantation trajectory.

## Figures and Tables

**Table 1:** Experimental estimation of the magnetic tracking accuracy.

Axis	X	Y	Z
Mean Absolute Error (mm)	1.76	2.00	0.30
Max Absolute Error (mm)	2.47	3.28	1.30

**Table 2:** Position discrepancies between magnetic tracking system and MR images.

Axis	X	Y	Z
Mean Absolute Distance (mm)	0.52	1.48	5.10
Max Absolute Distance (mm)	0.90	2.06	8.94

## Data Availability

Data is contained within the article.
